# Examining proximity to death and health care expenditure by disease: a Bayesian-based descriptive statistical analysis from the National Health Insurance database in Japan

**DOI:** 10.1186/s13561-021-00353-9

**Published:** 2022-01-10

**Authors:** Yuji Hiramatsu, Hiroo Ide, Atsuko Tsuchiya, Yuji Furui

**Affiliations:** 1 Institute for Future Initiatives, The University of Tokyo, 7-3-1, Hongo, Bunkyo-ku, Tokyo, 113-0033 Japan; 2 MCVP Division, AXA Life Insurance Co., Ltd, Tokyo, Japan; 3 Health and Welfare Department, Shizuoka Prefectural Government, Shizuoka, Japan

**Keywords:** Health care expenditure, Proximity to death, Disease, Bayes, Japan

## Abstract

**Background:**

Japan is one of the Organization for Economic Co-operation and Development (OECD) countries where population aging and increasing health care expenditures (HCE) are urgent issues. Recent studies have identified factors other than age, such as proximity to death and morbidity, as contributing factors to the increase in medical costs. It is important to assess HCE by disease and analyze their factors to estimate and improve future HCE.

**Methods:**

We extracted individual records spanning approximately 2 years prior to the death of persons aged 65 to 95 years from the National Health Insurance data in Japan, and used a Bayesian approach to decompose monthly HCE into five disease groups (circulatory, chronic kidney disease, neoplasms, respiratory, and others). The relationship between the proximity to death and the average HCE in each disease group was stratified by sex and age and analyzed using a descriptive statistical method similar to the two-part model.

**Results:**

The average HCE increased rapidly as death approached in most disease groups, but the increase-pattern differed greatly among disease groups, sex, and age groups. The effect of proximity to death on average HCE was small for chronic diseases, but large for lethal diseases. When stratified by age and sex, younger and male decedents tended to have higher average HCE, but the extent of this varied by disease group. The two-year cumulative average HCE for neoplasms in the 65–75 years age group was about six times larger than those in the 85–95 years age group.

**Conclusions:**

In Japan, it was suggested that disease, proximity to death, age, and sex may contribute to HCE. However, these factors interact in a complex manner, and it is important to analyze HCE by disease. In addition, preventing or delaying the severity of diseases with high medical burdens in younger people may be effective in reducing future terminal care costs. These findings have important implications for future projections and improvements of HCE.

**Supplementary Information:**

The online version contains supplementary material available at 10.1186/s13561-021-00353-9.

## Background

Currently, health care expenditures (HCE) are on the rise in most Organization for Economic Co-operation and Development (OECD) countries, and the upward trend in the ratio of HCE to GDP has continued for more than 30 years [1]. Population aging has long been identified as a factor in the increase in HCE [2], and the former is still underway in many countries [1]. For example, in Japan, the percentage of the total population aged 65 and over has increased from 23.0 to 28.4% over the past 10 years, from 2010 to 2019. Many studies have examined the relationship between age and HCE [3–7]. It has been pointed out that the ratio of end-of-life care expenditures for decedents to total healthcare costs is greater than the ratio of decedents to the total population [8, 9]. According to Lubitz et al. [8], in Medicare although decedents accounted for only 6% of the total population, end-of-life HCE accounted for 28% of the total annual HCE, most of which was concentrated within the months before death. This indicates the importance of analyzing end-of-life HCE and factors that influence them to estimate future healthcare costs. It has also been pointed out that end-of-life care, which includes medical care costs, decreases with increasing age [8–10], suggesting that we cannot simply conclude that aging leads to an increase in medical costs.

After much debate about the relationship between aging and HCE, a “red herring” hypothesis was proposed [11], that suggested that although age and HCE are positively correlated, proximity to death (PTD), rather than age itself, is the main factor driving HCE, and numerous articles related to this debate have been published [12–44]. While Zweifel et al. [11] found that PTD was an important factor in HCE and not age itself, Seshamani et al. [23] have reported that both PTD and age are important factors. They solved the problems of multicollinearity and inappropriate handling of records with no medical costs in the Heckman model adopted by Zweifel et al. [11] by adopting a two-part model. Other studies [15, 16, 32] have pointed out the same thing, that PTD only reduces the overestimation of the effect of age on HCE, and that both PTD and age are important.

Werblow et al. [13] decomposed HCE into all seven healthcare services (ambulatory care, prescriptions, hospitalization, outpatient care, nursing home care, home care, and other services) and examined the association between PTD or age and HCE for each service. They reported that age was not significant for HCE for most services, while PTD was significant. However, they also noted that age had a significant effect on HCE associated with long-term care services. They termed this “school of red herring” as the fact that the associations between PTD or age and HCE differed depending on whether health care services were long-term care related or not. Polder et al. [21] used health insurance data of 13.4% of the Dutch population to analyze the total costs of medical care and nursing care in the year before death by gender and the disease that caused death. Results showed that among the deceased, younger age was associated with higher cost of medical care, while older age was associated with higher cost of nursing care and higher cost of malignant neoplasms as a cause of death in both men and women, especially in the younger age group. When the correlation between age and HCE was examined by controlling for diseases that caused death, morbidity, number of comorbidities, length of hospital stay, and PTD, HCE was reported to be lower in older age groups [20, 22–28, 42]. The reasons for lower HCE in older age groups include nursing care services as a substitute function for medical services in the elderly and reduced medical intensity [18, 22, 23, 35]. Wong et al. [24] analyzed hospital HCE for 94 different diseases among Dutch survivors and decedents and found that the significance and effect of PTD and age differed for each disease, which they termed the “carpaccio of red herring.” Dormont et al. [14] also analyzed the relationship between aging, changes in morbidity and medical technology, and HCE, and concluded that the impact of aging on HCE is smaller than that of advanced medical care on HCE. In addition, some studies have reported that the closer one is to death, the more likely one is to have diseases, and that PTD itself is a substitute for morbidity [20, 27], suggesting that it is important to consider morbidities when analyzing the factors of HCE. Thus, although there have been many discussions until recently, no conclusion has been reached on the factors that determine HCE, and research is still being conducted that include not only age and PTD, but also the effects of lifestyle, income, price, and medical environment [37–41, 44].

Japan is an OECD country where population aging is serious and at the same time, the increase in HCE has become a problem. In the early 2000s, the percentage of the total population aged 65 years or older was less than 20%, but in 2019, it had reached 28% [1], and verifying the correlation between age and HCE is an important issue in estimating future healthcare costs in Japan. Hashimoto et al. [18] examined the relationship between age, PTD, and health care and nursing care expenditures using claims data from the National Health Insurance (NHI) of the population aged 65 and older in the Kyushu district of Japan. They pointed out the possibility of both the contribution of PTD to increased HCE and the contribution of aging to increased nursing care expenditures. Hosoya [19] used macroeconomic data of 25 OECD countries, including Japan, and estimated the relationship between age and HCE in a fixed effects model after controlling for other macroeconomic variables such as GDP.

In this study, to examine the relationship between PTD and HCE, we stratified our data by sex and age group and examined trends in average HCE for each month from the month of death to 23 months prior. In Japan, reimbursement claims data are summed up monthly and HCE by disease is not known, making it unclear how much medical expenses are spent on which diseases. Therefore, we used the Bayesian methods to decompose the incurred HCE into five representative disease groups and analyzed the average per capita HCE for each disease group for each month prior to death. Our proposed method for appropriate allocation of costs by disease is to obtain the average HCE of representative diseases while considering the uncertainty of the parameters that enables analysis of HCE by disease and is useful for understanding the trend of average HCE. Polder et al. [23] analyzed medical and care costs by the disease that caused death. In this study, however, we used our proposed method to allocate the incurred HCE to each disease group on an average basis. Ours is a novel method of analysis that is more objective in that it appropriately allocates costs to diseases other than those that cause death. In addition, due to the aforementioned problems with reimbursement claims data in Japan, there have been no studies on the relationship between PTD and HCE by disease [18], which is another novelty of this study.

## Methods

### Study objective

The purpose of this study was to clarify the relationship between PTD and end-of-life HCE for diseases using data from the Japanese National Health Insurance System. We analyzed the relationship between PTD and HCE, stratified by sex, age, and disease group, considering the results of studies outside Japan that have reported the importance of the relationship between morbidity and age [20–28]. The HCE for each stratified group were estimated for each month prior to death using a method similar to the two-part model that has been frequently used since Seshamani et al. [23, 33]. Finally, the estimated HCE were accumulated for approximately 2 years prior to death, and the impact of each disease group on the cost of medical care at the end of life was examined, and differences by sex, age, and disease were analyzed.

### Data

The data used in this study were information on enrollees and reimbursement claims data from the NHI in the Shizuoka Prefecture, located in the center of Japan. The NHI is a public medical insurance program that requires self-employed and retired elderly people to join. Information on enrollees is anonymized individual-level data, including demographic information such as date of birth, date of death, and gender. Reimbursement claims data are a record of monthly total HCE and correspond to ICD-10 codes, which can be linked to information on enrollees by anonymized IDs. The billed HCE consists of inpatient and outpatient expenditures and prescription fees, but it is not broken down by the disease. For the purpose of this study, it was necessary to estimate the average HCE for each of the five disease groups using the decomposition method of HCE, which will be explained later. The diseases were divided into five groups: circulatory (I00-I99), chronic kidney disease (N18), neoplasms (C00-D48), respiratory (J00-J99), and others according to the WHO ICD-10 definition [45]. The three major causes of death among the elderly (65 years and older) in Japan are neoplasms, heart disease, and cerebrovascular disease, in descending order of frequency. Heart and cerebrovascular diseases are included in the circulatory group in this study. Others consisted of all ICD-10 codes except for circulatory, Chronic Kidney Disease (CKD), neoplasms, and respiratory diseases. In the ICD-10 codes categorized as others, there were some disease groups such as diabetes mellitus and metabolic disorders that were attributed to lifestyle and were of interest in this study. However, in the decomposition method for HCE described below, some cases occurred where the estimation was not stable due to insufficient sample size, so they were included in others.

The NHI data recorded between November 2012 and October 2018 were used, and there were 4,292,759 samples including both surviving and deceased individuals. We selected insured individuals who had died during the study period, had at least 2 years of coverage, and were between 65 and 95 years of age at death. As a result, 122,318 samples were included in the analysis. The age at death was divided into three age groups: 65–75 years, 75–85 years, and 85–95 years. Seshamani et al. [33] pointed out that the effect of PTD becomes apparent 15 years before death, and Wyl [28] found that HCE increased significantly more than 2 years before death. In this study, the time to death (TTD) to be analyzed was 23 months (approximately 2 years), based on both the results of these previous studies and the sufficient data points required to decompose HCE in our method. Seshamani et al. [23] and Kolodziejczyk [34] pointed out the problem of bias due to right-censoring of survivors who did not die within the observation period, but this problem did not arise in this study because only decedents were included in the analysis.

### Study procedures

Zweifel et al. [11] estimated the association of PTD and age with HCE using a two-step Heckman model [46] and found that PTD was significant and had a large effect. However, Salas et al. [29] and Seshamani et al. [23, 33] pointed out multicollinearity due to the inverse Mills ratio calculated using Heckman’s method and the endogeneity of PTD and avoided the problem of multicollinearity by adopting a two-part model. The two-part model has been known for a long time in the field of actuaries who calculate insurance premium rates and is also called the “frequency-severity model” [47]. In the two-part model [48], the first step is to model the probability of incurring HCE (frequency), and the second is to model the HCE conditional on incurring IHCE (incurred health care expenditures). In this study, following Klugman et al. [47], we refer to the model of the first step as the frequency model and the model of the second step as the severity model. The average HCE (AHCE) can then be calculated by multiplying the estimated frequency by the IHCE [49]. Since frequency and IHCE are estimated by stratified group, TTD (in months), and disease group, AHCE also represents the estimated per capita by stratified group, TTD, and disease group.

In the frequency model, we defined the estimated frequency, *F*_.*gtd*_, as the proportion of HCE occurrences by stratified and disease groups in the corresponding TTD, as shown in the following equation:

$$ {\boldsymbol{F}}_{.\boldsymbol{gtd}}=\frac{\mathbf{1}}{{\boldsymbol{N}}_{\boldsymbol{g}}}\sum \limits_{\boldsymbol{i}=\mathbf{1}}^{{\boldsymbol{N}}_{\boldsymbol{g}}}{\boldsymbol{I}}_{\boldsymbol{i}\boldsymbol{gtd}} $$

$$ N=\sum \limits_{g=1}^G{N}_g $$

where *I*_*igtd*_ is a variable indicating whether or not the *i*-th subject in the *g*-th stratified group (1 ≤ *g* ≤ *G*) had HCE in disease group *d* (1 ≤ *d* ≤ 5, five types of disease group) in the month before death *t* (0 ≤ *t* ≤ 23), and is an indicator variable that takes the value of 1 if HCE is incurred and 0 otherwise. *G* represents the number of types of analysis targets stratified by sex, three age groups, or both, and the largest number of types is six, multiplied by the number of types of sex and age groups (*G* = 2 × 3 = 6). *N* is the total number of individuals analyzed, and *N*_*g*_ is the number of individuals belonging to the *g*-th stratified group.

By contrast, in the severity model, the IHCE of each individual is summed up by month, so that cost allocations need to be made to the five disease groups. In Japan, a method called the “primary disease method” is often used to estimate IHCE by disease. This method allocates all the IHCE in the month to the disease for which most medical resources are considered to have been invested and it has several drawbacks. First, this method depends on the subjectivity of the person who judges the primary disease. Second, all medical resources spent on other coexisting diseases are also allocated to the primary disease, resulting in a bias in the estimation of IHCE by disease. Therefore, a method that enables an objective and appropriate cost allocation is necessary.

Wagner et al. [50] used Ordinary Least Squares (OLS) regression to estimate medical costs by diagnostic group, but they reported cases in which the estimated medical costs were negative. This is an unavoidable problem because OLS assumes a normal distribution. On the other hand, Zweifel et al. [11] and many other authors solved the problem of negative HCE by logarithmically transforming HCE. However, when heteroscedasticity exists, it has been pointed out that bias occurs when retransforming to the original medical cost dimension, and Seshamani et al. [23] proposed a generalized linear model using the log-link function as an alternative.

**Fig. 1:**
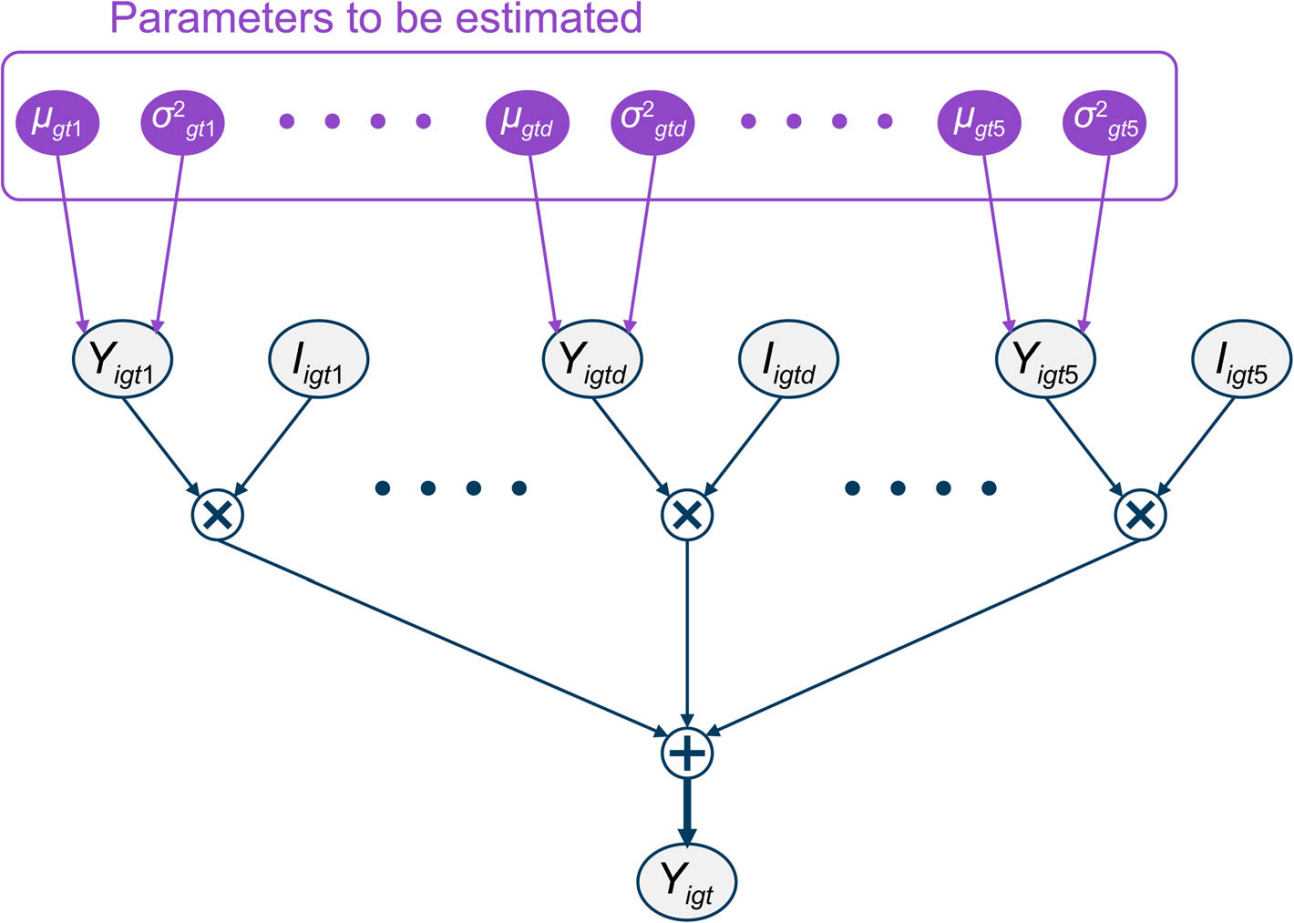
Network graph representing the Severity Model

The IHCE of the same disease may vary depending on the hospital where the patient receives treatment, doctor’s judgment, and the comorbidity, and the mean value of IHCE may not be truly one value. In addition, if the covariates that represent these factors are not sufficiently obtained as data, the methods described thus far are not necessarily optimal, and fitting, including the uncertainty of the estimated values, is important. Furthermore, the HCE is a sum of normal distributions with different model parameters, and the Bayesian method using Markov Chain Monte Carlo (MCMC) is suitable for estimating the model parameters of each normal distribution in such cases. And by adopting the method using MCMC, the expected value of medical cost for each disease did not become negative as seen in Wagner et al. [50]. Figure 1 shows a network graph of the relationship between the parameters to be estimated and the summed IHCE *t* months before the death of the *i*-th subject belonging to the stratified group *g*.

*Y*_*igt*_ in Fig. 1 is the IHCE of the *i*-th subject in the stratified group *g* at *t* months before death and is an observable value (gray in Fig. 1 indicates that it is an observable value). *I*_*igtd*_ in Fig. 1 is an indicator variable that specifies whether the *i*-th subject in group *g* has or does not have IHCE for disease group *d* at *t* months before death and is the same variable as *I*_*igtd*_ in the frequency model. *Y*_*igtd*_ in Fig. 1 is a random variable representing the IHCE of disease group *d* at *t* months before the death of the *i*-th subject, following a normal distribution with mean value *μ*_*gtd*_ and variance *σ*^2^_*gtd*_, assuming that IHCE of each disease group is independent of each other. The purple line in Fig. 1 is the parameter to be estimated, while the variance *σ*^2^_*gtd*_ is a nuisance parameter and is not of interest in this study. The structure shown in Fig. 1 can be expressed using the following equation:

$$ {Y}_{igt}=\sum \limits_{d=1}^5{Y}_{igt d}\bullet {I}_{igt d} $$

$$ {Y}_{igtd}\sim Normal\ \left({\mu}_{gt d},{\sigma}_{gt\mathrm{d}}^2\right) $$

We used R’s RStan 2.19.3 [51] to estimate the posterior distributions of the parameters (*μ*_*gtd*_, *σ*^2^_*gtd*_) by sampling with MCMC. With reference to the mean and variance in the sample in each stratified group of IHCE, a weakly informative prior distributions were adopted as the prior distributions for each parameter so that the calculation would converge efficiently. HCE was normalized to 100,000 Japanese yen (JPY), and all subsequent numbers related to medical costs in this study are shown as normalized values (1 JPY is equivalent to 0.0093 USD at the average exchange rate during the sample period). The prior distributions of the parameters were the same for all disease groups and stratified groups, as follows:

$$ {\mu}_{gtd}\sim Normal\ \left(0,{20}^2\right) $$

$$ {\sigma}_{gtd}^2\sim LogNormal\ \left(0,{10}^2\right) $$

where *LogNormal* represents the lognormal distribution, and this distribution is chosen such that the variance does not take a negative value. For MCMC sampling, the number of chains was set to four, and the number of samples in each chain was set to 6000. The first 2000 steps were discarded as a warm-up period. The convergence condition of MCMC was set as R̂ of all parameters and log posterior probability being less than 1.05 [52], and convergence was confirmed in all calculations. Since Bayesian methods were used to analyze the IHCE, Bayesian credible intervals are reported in this paper instead of *P* values. In addition, the Bayes factor (BF) [53] for the composite hypothesis was used to verify whether there was a difference in IHCE by sex and age group, and the evidence criteria expressed in Table 1 based on Kass et al. [54] were adopted in this study.

**Table 1:** Evidence criteria for Bayes Factor

Bayes Factor	Level of evidence
1 to 3	Not worth more than a bare mention
3 to 20	Positive
20 to 150	Strong
> 150	Very strong

Finally, based on the results obtained from the frequency and severity models, the average HCE (AHCE) for each disease group and the cumulative average HCE (CAHCE) over a period of approximately 2 years before death were calculated [49]. The formulation is as follows:

$$ {AHCE}_{gtd}={\hat{\mu}}_{gtd}\bullet {F}_{. gtd} $$

$$ C{AHCE}_{gd}=\sum \limits_{t=0}^{23}{AHCE}_{gtd} $$

where ${\hat{\mu}}_{gtd}$ denotes the mean value of the posterior distribution of *μ*_*gtd*_, *AHCE*_*gtd*_ denotes the AHCE of disease group *d* in stratified group *g* at *t* months before death, and *CAHCE*_*gd*_ denotes the CAHCE of disease group *d* in the stratified group.

### Statistical analysis

We classified age into three groups and stratified the subjects into up to six categories based on age group and sex. For each stratified category, a frequency model and severity model were created for each of the five disease groups for each TTD, and finally, AHCE and CAHCE were calculated for each disease group.

To verify whether the differences in the values of frequency for each category were significant, the Chi-square test was conducted for each category and the number of HCE incurred. We also performed the Kruskal-Wallis and the Wilcoxon tests with Bonferroni correction to test whether the difference in total IHCE, the sum of the IHCE of the five disease groups, between the age groups was significant. Similarly, the difference in total IHCE between the two sexes was analyzed using the Wilcoxon test. Pearson’s correlation coefficient was calculated for each TTD to check whether the IHCE of each disease group were independent of each other. The decomposed IHCE by disease group was then tested for differences between sex or age groups by Bayes factor.

The above analysis was performed by extracting the relevant data for each subject for each month from 0 to 23 months prior to death.

## Results

### Basic statistics

The number of individuals used in this analysis was 122,318 who were stratified by sex, age group, or both to analyze the relationship between PTD and HCE for each disease group. Table 2 shows the number of individuals analyzed by sex and age group. The number of males and females was almost the same, but the proportion of the older adults was larger in females, which is attributed to their longer life expectancy.

**Table 2:** Number of subjects by sex and age group

	Male			Female			All
Age	65–75	75–85	85–95	65–75	75–85	85–95	
*N*	10,699	25,165	27,630	5154	16,783	36,887	122,318
Ratio (%)	8.7%	20.6%	22.6%	4.2%	13.7%	30.2%	100%

Table 3 shows the number of HCE incurred for each disease group when TTD is 0 to 6 months (TTD of 7 or more is not shown). Table 4 shows the mean total IHCE, which was the sum of IHCE for all diseases when the TTD is 0 to 6 months. As a general trend, the number of HCE incurred was larger in the following order: others, circulatory, respiratory, neoplasms, and CKD. The number of HCE incurred in the others group was the largest because it included a majority of the diseases. Regarding the month of death (TTD = 0), there was a downward trend in the number of HCE incurred and total IHCE compared with the month before death (TTD = 1), because the effective period in the month of death was only half a month. Despite this, the number of respiratory cases was characteristically higher than that in the month before death. Table 3 shows only the number of HCE incurred with TTD from 0 to 6, but values with TTD from 7 to 23 were also included in the calculation of Frequency.

**Table 3:** Number of HCE (health care expenditures) incurred for each disease group

			Time to death						
Sex	Age Group	Disease Group	6	5	4	3	2	1	0
Male	65–75	Circulatory^1^	5632 (4.6%)	5717 (4.7%)	5759 (4.7%)	5817 (4.8%)	5870 (4.8%)	5910 (4.8%)	5354 (4.4%)
		Respiratory	3531 (2.9%)	3610 (3.0%)	3792 (3.1%)	4028 (3.3%)	4251 (3.5%)	4732 (3.9%)	4932 (4.0%)
		CKD	691 (0.6%)	710 (0.6%)	723 (0.6%)	741 (0.6%)	758 (0.6%)	780 (0.6%)	698 (0.6%)
		Neoplasms	3728 (3.0%)	3891 (3.2%)	4116 (3.4%)	4327 (3.5%)	4647 (3.8%)	4990 (4.1%)	4784 (3.9%)
		Others	7850 (6.4%)	7935 (6.5%)	8040 (6.6%)	8181 (6.7%)	8314 (6.8%)	8547 (7.0%)	7983 (6.5%)
	75–85	Circulatory	14,111 (11.5%)	14,144 (11.6%)	14,232 (11.6%)	14,130 (11.6%)	14,206 (11.6%)	14,164 (11.6%)	12,744 (10.4%)
		Respiratory	8521 (7.0%)	8850 (7.2%)	9087 (7.4%)	9503 (7.8%)	10,333 (8.4%)	11,574 (9.5%)	11,936 (9.8%)
		CKD	1881 (1.5%)	1909 (1.6%)	1950 (1.6%)	1985 (1.6%)	2081 (1.7%)	2154 (1.8%)	1889 (1.5%)
		Neoplasms	6000 (4.9%)	6242 (5.1%)	6561 (5.4%)	6863 (5.6%)	7376 (6.0%)	8012 (6.6%)	7499 (6.1%)
		Others	17,243 (14.1%)	17,309 (14.2%)	17,401 (14.2%)	17,481 (14.3%)	17,727 (14.5%)	18,055 (14.8%)	16,635 (13.6%)
	85–95	Circulatory	19,551 (16.0%)	19,613 (16.0%)	19,621 (16.0%)	19,722 (16.1%)	19,894 (16.3%)	20,054 (16.4%)	18,409 (15.1%)
		Respiratory	11,504 (9.4%)	11,832 (9.7%)	12,396 (10.1%)	13,119 (10.7%)	14,295 (11.7%)	16,263 (13.3%)	16,891 (13.8%)
		CKD	2252 (1.8%)	2318 (1.9%)	2341 (1.9%)	2399 (2.0%)	2490 (2.0%)	2652 (2.2%)	2402 (2.0%)
		Neoplasms	5811 (4.8%)	6005 (4.9%)	6311 (5.2%)	6662 (5.4%)	7198 (5.9%)	8045 (6.6%)	7598 (6.2%)
		Others	23,004 (18.8%)	23,009 (18.8%)	23,212 (19.0%)	23,451 (19.2%)	23,725 (19.4%)	24,218 (19.8%)	22,415 (18.3%)
Female	65–75	Circulatory	2909 (2.4%)	2887 (2.4%)	2902 (2.4%)	2933 (2.4%)	2924 (2.4%)	2996 (2.4%)	2758 (2.3%)
		Respiratory	1747 (1.4%)	1741 (1.4%)	1783 (1.5%)	1840 (1.5%)	1990 (1.6%)	2223 (1.8%)	2264 (1.9%)
		CKD	299 (0.2%)	310 (0.3%)	311 (0.3%)	313 (0.3%)	335 (0.3%)	343 (0.3%)	302 (0.2%)
		Neoplasms	1967 (1.6%)	2024 (1.7%)	2103 (1.7%)	2241 (1.8%)	2377 (1.9%)	2547 (2.1%)	2447 (2.0%)
		Others	4193 (3.4%)	4232 (3.5%)	4263 (3.5%)	4329 (3.5%)	4371 (3.6%)	4462 (3.6%)	4111 (3.4%)
	75–85	Circulatory	11,033 (9.0%)	11,051 (9.0%)	11,105 (9.1%)	11,143 (9.1%)	11,158 (9.1%)	11,194 (9.2%)	10,387 (8.5%)
		Respiratory	5611 (4.6%)	5704 (4.7%)	5870 (4.8%)	6195 (5.1%)	6628 (5.4%)	7495 (6.1%)	8043 (6.6%)
		CKD	1087 (0.9%)	1108 (0.9%)	1106 (0.9%)	1178 (1.0%)	1187 (1.0%)	1236 (1.0%)	1106 (0.9%)
		Neoplasms	3631 (3.0%)	3793 (3.1%)	4013 (3.3%)	4267 (3.5%)	4635 (3.8%)	5046 (4.1%)	4845 (4.0%)
		Others	13,841 (11.3%)	13,866 (11.3%)	13,955 (11.4%)	14,092 (11.5%)	14,224 (11.6%)	14,366 (11.7%)	13,278 (10.9%)
	85–95	Circulatory	25,739 (21.0%)	25,771 (21.1%)	25,897 (21.2%)	25,975 (21.2%)	26,249 (21.5%)	26,392 (21.6%)	24,882 (20.3%)
		Respiratory	11,502 (9.4%)	11,864 (9.7%)	12,365 (10.1%)	13,070 (10.7%)	14,274 (11.7%)	16,441 (13.4%)	17,989 (14.7%)
		CKD	1847 (1.5%)	1888 (1.5%)	1969 (1.6%)	2024 (1.7%)	2110 (1.7%)	2278 (1.9%)	2124 (1.7%)
		Neoplasms	4082 (3.3%)	4318 (3.5%)	4643 (3.8%)	5018 (4.1%)	5670 (4.6%)	6395 (5.2%)	6309 (5.2%)
		Others	29,713 (24.3%)	29,855 (24.4%)	30,091 (24.6%)	30,140 (24.6%)	30,713 (25.1%)	31,000 (25.3%)	28,844 (23.6%)

^1^The figures in parentheses indicate the percentage of the number of cases to the total number of subjects

*CKD* Chronic Kidney Disease

**Table 4:** Mean total IHCE (incurred health care expenditures)

		Time to death						
Sex	Age Group	6	5	4	3	2	1	0
Male	65–75	2.9 (4.3)	3.2 (4.7)	3.4 (4.7)	3.8 (5.4)	4.3 (5.7)	5.7 (6.3)	4.4 (6.0)
	75–85	2.2 (3.6)	2.5 (4.0)	2.7 (4.1)	3.1 (4.5)	3.7 (4.6)	4.9 (5.4)	3.7 (4.7)
	85–95	1.7 (3.0)	1.9 (3.0)	2.1 (3.2)	2.5 (3.5)	3.1 (3.8)	4.2 (4.3)	3.1 (3.5)
Female	65–75	2.9 (4.4)	3. (4.2)	3.1 (4.4)	3.5 (4.9)	4.3 (5.5)	5.6 (7.3)	4.3 (6.1)
	75–85	2.1 (3.3)	2.3 (3.9)	2.5 (3.9)	2.8 (4.2)	3.4 (4.4)	4.5 (5.6)	3.5 (5.0)
	85–95	1.5 (2.6)	1.6 (2.7)	1.8 (3.1)	2.1 (3.3)	2.5 (3.5)	3.3 (3.8)	2.5 (3.1)

^1^The unit of IHCE (incurred health care expenditures) is 100,000 JPY

^2^The figures in parentheses indicate the standard deviation of total IHCE (incurred health care expenditures)

Table 5 shows the admission rate based on TTD. The admission rate increased rapidly as the month of death approached. The older the group, the lower the admission rate tended to be across all TTD.

**Table 5:** Admission ratio by time to death

		Time to death						
Sex	Age Group	6	5	4	3	2	1	0
Male	65–75	18.1%	19.9%	22.0%	25.6%	31.8%	43.6%	69.8%
	75–85	16.8%	19.0%	21.6%	25.9%	32.5%	44.2%	67.2%
	85–95	15.1%	17.3%	20.1%	24.6%	31.6%	43.2%	63.2%
Female	65–75	16.9%	18.6%	20.4%	23.6%	30.4%	41.8%	67.5%
	75–85	18.0%	20.0%	22.4%	25.9%	31.6%	42.0%	63.2%
	85–95	15.4%	17.0%	19.3%	22.6%	28.0%	36.7%	53.0%

Table 6 shows the results of the Chi-square test for the age group being independent of the number of HCE incurred (the number of records with non-zero HCE) for each disease group with TTD from 0 to 6 months, based on the number of HCE incurred in Table 3. While there was a significant relationship between age group and the number of HCE incurred for most of the disease groups, there were several TTD for which there was no significant relationship with others. Table 7 shows the results of the Chi-square test for the independence between sex and the number of HCE incurred for each disease group with TTD from 0 to 6 months, based on the number of HCE incurred in Table 3. The relationship between sex and the number of HCE incurred tended to become more significant in older age across all disease groups.

**Table 6:** Chi-square test for the age group being independent of the number of HCE (health care expenditures) incurred

		Time to death						
Sex	Disease Group	6	5	4	3	2	1	0
Male	Circulatory	0.000***	0.000***	0.000***	0.000***	0.000***	0.000***	0.000***
	Respiratory	0.000***	0.000***	0.000***	0.000***	0.000***	0.000***	0.000***
	CKD	0.000***	0.000***	0.000***	0.000***	0.000***	0.000***	0.000***
	Neoplasms	0.000***	0.000***	0.000***	0.000***	0.000***	0.000***	0.000***
	Others	0.018*	0.346	0.087	0.484	0.375	0.023*	0.076
Female	Circulatory	0.000***	0.000***	0.000***	0.000***	0.000***	0.000***	0.000***
	Respiratory	0.000***	0.011*	0.171	0.144	0.321	0.000***	0.000***
	CKD	0.000***	0.000***	0.000***	0.000***	0.000***	0.000***	0.003**
	Neoplasms	0.000***	0.000***	0.000***	0.000***	0.000***	0.000***	0.000***
	Others	0.000***	0.217	0.008**	0.052	0.026*	0.091	0.000***

*p < 0.05, **p < 0.01, ***p < 0.001

*CKD* Chronic Kidney Disease

**Table 7:** Chi-square test for the independence of sex and the number of HCE (health care expenditures) incurred

		Time to death						
Age Group	Disease Group	6	5	4	3	2	1	0
65–75	Circulatory	0.009**	0.000***	0.000***	0.000***	0.000***	0.029*	0.753
	Respiratory	0.001***	0.000***	0.000***	0.000***	0.000***	0.000***	0.000***
	CKD	0.002**	0.003**	0.002**	0.001***	0.007**	0.006**	0.007**
	Neoplasms	0.581	0.279	0.066	0.314	0.143	0.185	0.483
	Others	0.848	0.162	0.448	0.198	0.367	0.712	0.277
75–85	Circulatory	0.000***	0.000***	0.000***	0.005**	0.004**	0.381	0.000***
	Respiratory	0.000***	0.000***	0.000***	0.000***	0.000***	0.000***	0.000***
	CKD	0.000***	0.000***	0.000***	0.000***	0.000***	0.000***	0.000***
	Neoplasms	0.000***	0.000***	0.000***	0.000***	0.000***	0.000***	0.000***
	Others	0.000***	0.000***	0.000***	0.000***	0.001***	0.305	0.265
85–95	Circulatory	0.000***	0.000***	0.000***	0.000***	0.000***	0.000***	0.000***
	Respiratory	0.000***	0.000***	0.000***	0.000***	0.000***	0.000***	0.000***
	CKD	0.000***	0.000***	0.000***	0.000***	0.000***	0.000***	0.000***
	Neoplasms	0.000***	0.000***	0.000***	0.000***	0.000***	0.000***	0.000***
	Others	0.000***	0.000***	0.000***	0.000***	0.000***	0.000***	0.001***

*p < 0.05, **p < 0.01, ***p < 0.001

*CKD* Chronic Kidney Disease

Total IHCE was lower in older age groups, with a significant difference in the Kruskal-Wallis test for TTD between 0 and 6 months (*p* < 0.001). The more conservative Wilcoxon test with Bonferroni correction also showed a significant difference among all age groups (*p* < 0.001). Conversely, in both 75–85 and 85–95 age groups, the Wilcoxon test showed significant differences in total IHCE between males and females at TTD of 0 to 6 months (*p* < 0.001), but in the 65–75 age group, there were no significant differences in most TTD (Table 8).

**Table 8:** Wilcoxon test in total IHCE (incurred health care expenditures) between men and women

	Time to death						
Age Group	6	5	4	3	2	1	0
65–75	0.481	0.095	0.003**	0.011*	0.332	0.045*	0.071
75–85	0.000***	0.000***	0.000***	0.000***	0.000***	0.000***	0.000***
85–95	0.000***	0.000***	0.000***	0.000***	0.000***	0.000***	0.000***

*p < 0.05, **p < 0.01, ***p < 0.001

Tables 9 and 10 show Pearson’s correlation coefficients of HCE occurrence for each disease group at TTD of 1 and 6 months. For most of the other TTD, there was not a strong correlation between the disease groups, suggesting that the probability of incurring HCE for each disease group was almost independent of each other, but when the TTD was 1, there was a tendency for circulatory and neoplasms not to co-occur (*r* = − 0.12).

**Table 9:** Pearson correlation coefficients of health care expenditure occurrence at time to death of 1

	CKD	Neoplasms	Others	Respiratory
Circulatory	0.11	−0.12	0.08	0.09
CKD		−0.04	0.02	0.02
Neoplasms			−0.01	−0.01
Others				0.02

*CKD* Chronic Kidney Disease

**Table 10:** Pearson correlation coefficients of health care expenditure occurrence at time to death of 6

	CKD	Neoplasms	Others	Respiratory
Circulatory	0.11	−0.06	0.05	0.10
CKD		−0.01	0.02	0.03
Neoplasms			0.00	0.06
Others				0.00

*CKD* Chronic Kidney Disease

### Frequency and severity models

The results of the four types of analysis have been described in the following order: without stratification by age group and sex, with stratification by age group, with stratification by sex, and with stratification by both sex and age group.

#### Without stratification by age group and sex

Additional file 1 shows the frequency (a) and IHCE (b). The IHCE graph shows a 95% Bayesian confidence interval with a pale-colored band. Since the month of death has an effective period of only about half a month, the values of both frequency and IHCE decrease in the month of death for most of the disease groups. For all disease groups, the frequency tended to increase as the time of death approached, with a particularly large increase in the months prior to death in respiratory diseases, where frequency and IHCE 1 month before death were approximately 1.5 and 3.5 times higher than 12 months before death, respectively. This is a large value compared to other diseases and may represent the fact that many patients are subjected to medical treatment, such as intubation of a ventilator, as death approaches. In addition, except for others, the frequency was large for all TTD in the circulatory group, which may indicate that many diseases in the circulatory group are chronic. In IHCE, CKD had the highest medical costs for all TTD, except in the months immediately before death, probably because patients with CKD were on regular dialysis. Neoplasms were the next largest group, with relatively high medical costs across all TTD. The lowest IHCE in the circulatory group could be because most circulatory diseases are chronic, and the main medical cost involves prescriptions for antihypertensive drugs, which are less expensive than the treatment cost of lethal diseases such as neoplasms. In all disease groups, IHCE tended to increase rapidly in a nonlinear pattern in the months before death, except in the month of death.

Additional file 2 shows AHCE (a) and CAHCE (b) for each disease group. Comparing AHCE by disease group, the order of increasing AHCE is others, respiratory, neoplasms, CKD, and circulatory. The AHCE in the others group increased about 2.5-fold when compared with the AHCE 1 year before death and 1 month before death, but the degree of increase differed among disease groups, and there was almost no change in chronic diseases (CKD and circulatory). Although the frequency of circulation is high, it is not large in terms of AHCE due to the small IHCE, but the difference in AHCE between groups tended to disappear as TTD increased. In the CAHCE, others accounted for approximately 60% of the total CAHCE (3,200,000 JPY in 2 years), and respiratory and neoplasms each accounted for approximately 15%.

Table 11 shows a comparison of the estimated total average HCE and the corresponding actual total HCE for each TTD. The error rate is the difference between the total average HCE and the actual total HCE divided by the actual total HCE, indicating that the AHCE can be estimated with good accuracy (less than ±0.3%) across all TTD.

**Table 11:** Comparison of the estimated total average HCE (health care expenditures) with the corresponding actual values

Time to death (Month)	Actual Total HCE (100,000 JPY)	Total Average HCE (100,000 JPY)	Error Rate (%)
0	360,495	360,494	0.00%
1	497,861	499,327	−0.29%
2	367,603	368,625	−0.28%
3	301,841	302,223	−0.13%
4	261,238	261,404	−0.06%
5	235,214	235,383	−0.07%
6	214,443	214,495	−0.02%
7	199,898	199,943	−0.02%
8	190,633	190,707	−0.04%
9	183,131	183,189	−0.03%
10	175,133	175,153	−0.01%
11	166,136	166,148	−0.01%
12	160,428	160,438	−0.01%
13	154,892	154,902	−0.01%
14	151,393	151,395	0.00%
15	147,516	147,522	0.00%
16	142,616	142,628	−0.01%
17	137,561	137,547	0.01%
18	135,438	135,440	0.00%
19	132,573	132,577	0.00%
20	129,362	129,356	0.00%
21	127,710	127,707	0.00%
22	123,939	123,940	0.00%
23	121,760	121,753	0.01%
SUM	4,818,815	4,822,295	−0.07%

*HCE*, health care expenditures

#### With stratification by age group

**Fig. 2:**
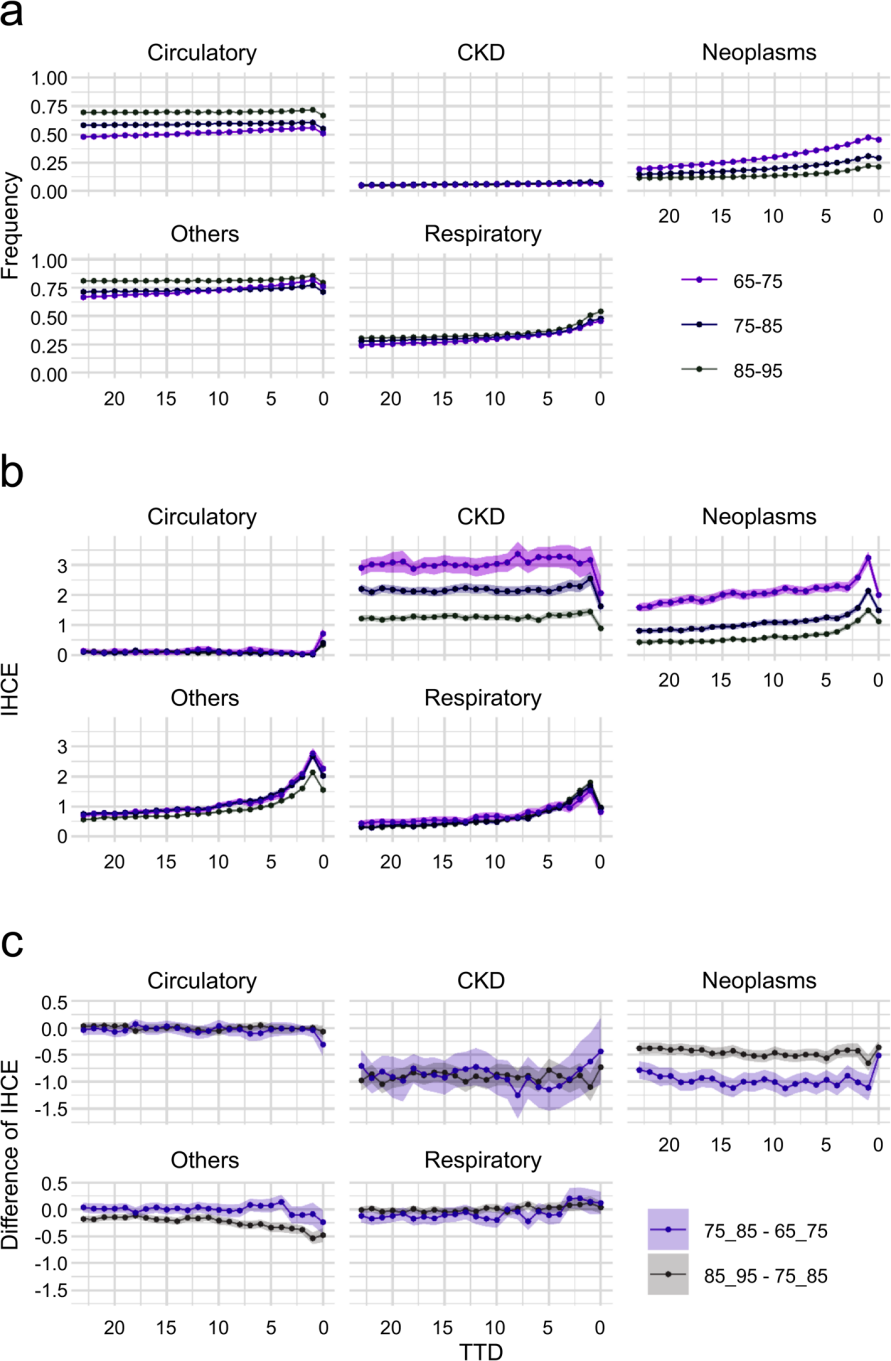
Frequency and IHCE (incurred health care expenditures) with stratification by age group

**Fig. 3:**
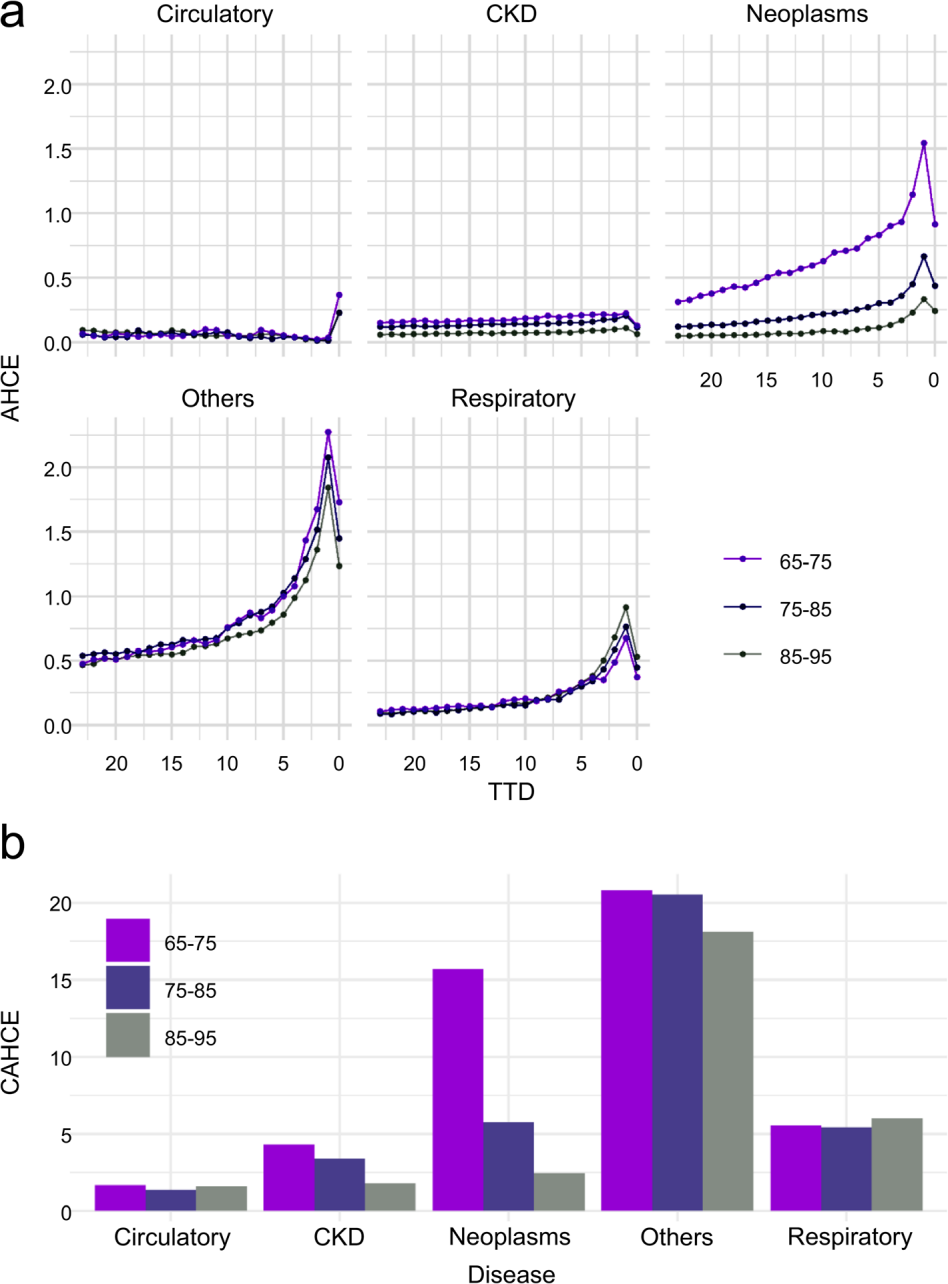
AHCE (average health care expenditures) and CAHCE (cumulative average health care expenditures) with stratification by age group

Figure 2 shows the estimation results by age group. Figure 2c shows the difference in posterior distributions between the 85–95 and 75–85 age groups, and between the 75–85 and 65–75 age groups by TTD, and the pale bands indicate the 95% Bayesian credible intervals. The frequency was higher in the older age group for all TTD in the circulatory and respiratory systems, and in others from the month of death to several months before death. In neoplasms, the frequency tended to be higher in the younger age group. In IHCE, BF was calculated from the difference in posterior distributions between the age groups of 85–95 years and 75–85 years, and between the age groups of 75–85 years and 65–75 years, to verify the strength of evidence between the age groups. Table 12 shows the results of calculating the BF values for each disease group by TTD (from 0 to 12 months) among the age groups. According to the results, the difference in IHCE in others, CKD, and neoplasms between the age groups of 85–95 years and 75–85 years was considered strong evidence, and IHCE was higher in the age group of 75–85 years; however, differences in circulatory and respiratory rates were not found across all TTD. Conversely, between the age groups of 75–85 years and 65–75 years, there was strong evidence of a difference in CKD and neoplasms, and IHCE was higher in the age group of 65–75 years, but there was no difference across all TTD for the other disease groups. Figure 3 shows the AHCE and CAHCE for each disease group by age group. In general, the younger age group had larger values for both AHCE and CAHCE, but the older age group tended to have larger values for respiratory diseases. In neoplasms, AHCE and CAHCE were remarkably larger in the 65–75 years age group than in other age groups, and the effect of PTD was also large. In particular, the CAHCE of the of 65–75 years age group was about three times larger than that of the of 75–85 years age group and about six times larger than that of the of 85–95 years age group.

**Table 12:** Bayes Factors for IHCE (incurred health care expenditures) difference with stratification by age group

TTD	Age	Circulatory	Respiratory	Others	CKD	Neoplasms
0	85–95 – 75–85	6.7*	3.1*	> 150 ***	> 150 ***	> 150 ***
0	75–85 – 65–75	> 150 ***	6.7*	54.7**	10.9*	> 150 ***
1	85–95 – 75–85	1.4	30.6**	> 150 ***	> 150 ***	> 150 ***
1	75–85 – 65–75	3.4*	8.1*	3.5*	95.4**	> 150 ***
2	85–95 – 75–85	1.6	18.3*	> 150 ***	> 150 ***	> 150 ***
2	75–85 – 65–75	1.9	39.6**	7.7*	> 150 ***	> 150 ***
3	85–95 – 75–85	1.8	11.9*	> 150 ***	> 150 ***	> 150 ***
3	75–85 – 65–75	1.2	53.2**	8.7*	> 150 ***	> 150 ***
4	85–95 – 75–85	1.3	3.8*	> 150 ***	> 150 ***	> 150 ***
4	75–85 – 65–75	1.2	5.6*	50.6**	> 150 ***	> 150 ***
5	85–95 – 75–85	1.1	3.3*	> 150 ***	> 150 ***	> 150 ***
5	75–85 – 65–75	1.6	8.7*	6.7*	> 150 ***	> 150 ***
6	85–95 – 75–85	9.0*	1.8	> 150 ***	> 150 ***	> 150 ***
6	75–85 – 65–75	12.5*	2.1	6.4*	> 150 ***	> 150 ***
7	85–95 – 75–85	1.6	56.8**	> 150 ***	> 150 ***	> 150 ***
7	75–85 – 65–75	13.4*	> 150 ***	12.6*	> 150 ***	> 150 ***
8	85–95 – 75–85	1.9	2.1	> 150 ***	> 150 ***	> 150 ***
8	75–85 – 65–75	2.0	2.5	1.7	> 150 ***	> 150 ***
9	85–95 – 75–85	1.1	5.5*	> 150 ***	> 150 ***	> 150 ***
9	75–85 – 65–75	1.9	1.0	2.2	> 150 ***	> 150 ***
10	85–95 – 75–85	8.9*	2.3	> 150 ***	> 150 ***	> 150 ***
10	75–85 – 65–75	2.7	> 150 ***	1.2	> 150 ***	> 150 ***
11	85–95 – 75–85	13.0*	3.1*	> 150 ***	> 150 ***	> 150 ***
11	75–85 – 65–75	3.8*	113.3**	1.5	> 150 ***	> 150 ***
12	85–95 – 75–85	3.5*	5.6*	> 150 ***	> 150 ***	> 150 ***
12	75–85 – 65–75	11.4*	25.9**	4.2*	> 150 ***	> 150 ***

*Positive (3 < BF < 20), **Strong (20 < BF < 150), ***Very strong (150 < BF)

*TTD* time to death, *CKD* Chronic Kidney Disease

#### With stratification by sex

**Fig. 4:**
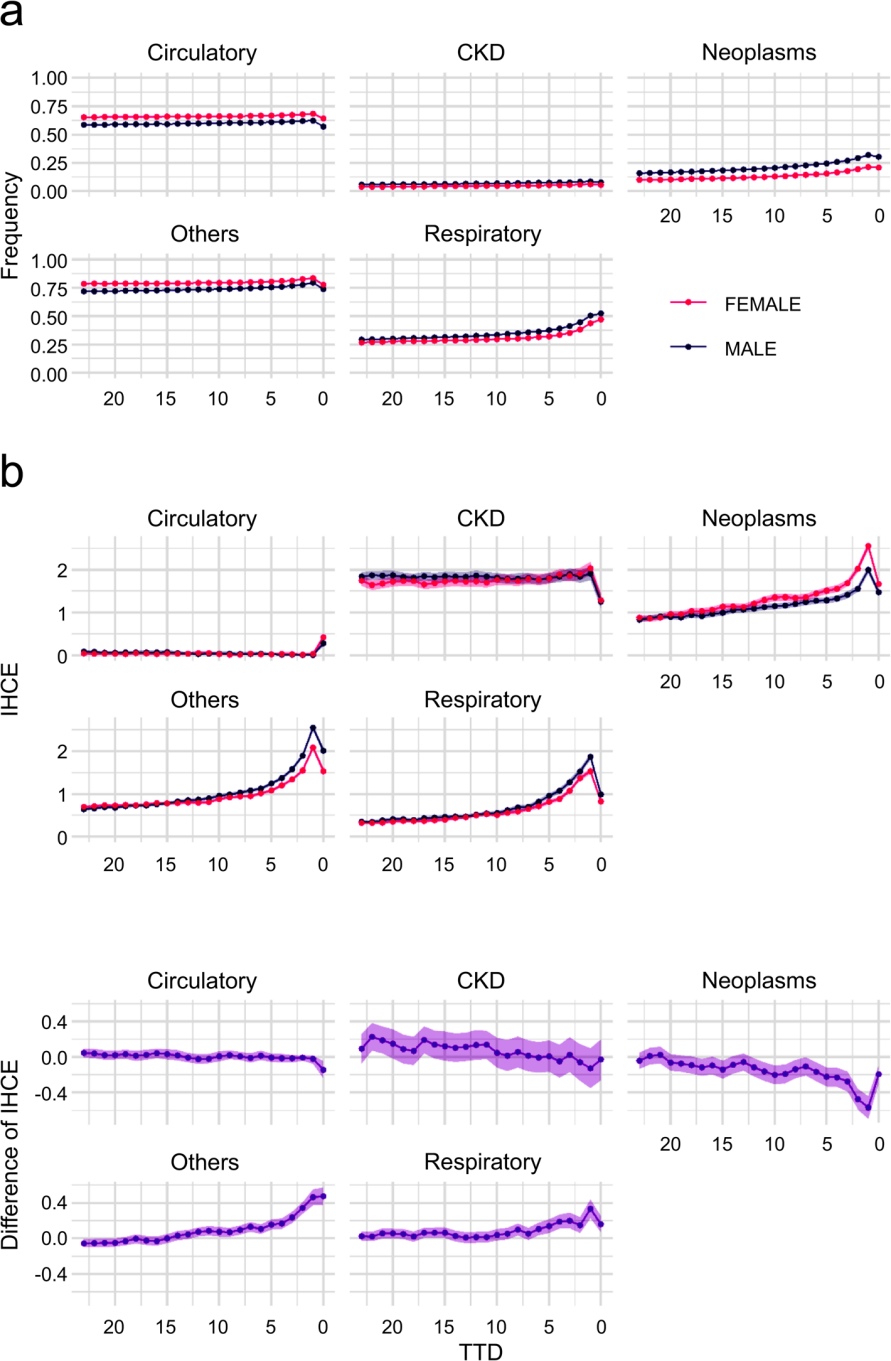
Frequency and IHCE (incurred health care expenditures) with stratification by gender

**Fig. 5:**
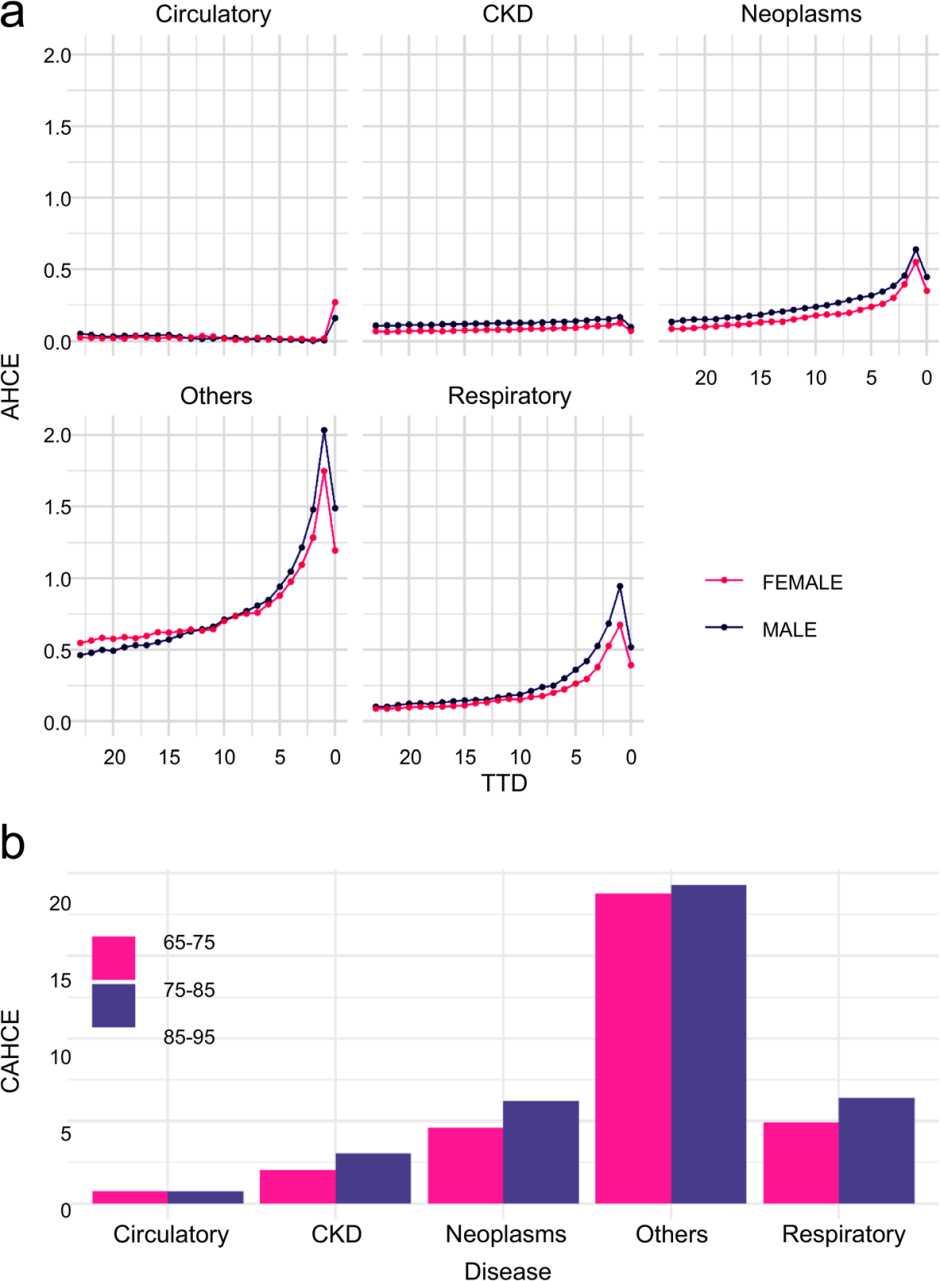
AHCE (average health care expenditures) and CAHCE (cumulative average health care expenditures) with stratification by gender

Figure 4 shows the estimation results by sex. The bottom graph (c) shows the difference in posterior distributions by TTD for males and females and the pale bands indicate the 95% Bayesian credible intervals. The frequency was higher for females in the circulatory and other groups, whereas it was higher for males in other disease groups. In the IHCE, BF was calculated from the difference in posterior distributions between males and females, and the strength of the evidence for the difference between males and females was tested. Table 13 shows the BF values for each TTD (from 0 to 12 months) for each disease group. The results show that there is no strong evidence of a difference between males and females in circulatory and CKD, but a difference was seen between males and females in respiratory, others, and neoplasms. For respiratory and others, IHCE was greater in males, but for neoplasms, IHCE was greater in females. Overall, the differences between the sexes were not as large as the differences between the age groups. Figure 5 shows the AHCE and CAHCE for each disease group by sex; males tended to have higher values for both.

**Table 13:** Bayes Factors for IHCE (incurred health care expenditures) difference with stratification by sex

TTD (Month)	Circulatory	Respiratory	Others	CKD	Neoplasms
0	> 150 ***	> 150 ***	> 150 ***	1.5	> 150 ***
1	4.5*	> 150 ***	> 150 ***	6.5*	> 150 ***
2	2.1	> 150 ***	> 150 ***	2.5	> 150 ***
3	3.0*	> 150 ***	> 150 ***	1.5	> 150 ***
4	2.2	> 150 ***	> 150 ***	2.3	> 150 ***
5	1.7	> 150 ***	> 150 ***	1.1	> 150 ***
6	2.6	> 150 ***	> 150 ***	1.1	> 150 ***
7	1.9	14.8*	> 150 ***	1.3	42.2**
8	1.9	> 150 ***	> 150 ***	2.9	> 150 ***
9	4.4*	18.7*	> 150 ***	1.3	> 150 ***
10	1.6	8.5*	> 150 ***	2.4	> 150 ***
11	2.9	2.2	> 150 ***	21.0**	> 150 ***
12	4.4*	2.1	> 150 ***	16.1*	68.9**

*Positive (3 < BF < 20), **Strong (20 < BF < 150), ***Very strong (150 < BF)

*TTD* time to death, *CKD* Chronic Kidney Disease

#### By age and sex

Additional file 3 shows the AHCE and CAHCE obtained by stratifying by age group and sex and applying the frequency and severity models (the results for frequency and IHCE are not shown). Except in respiratory diseases, CAHCE was larger in females than in males between the ages of 65 and 85. In the respiratory, CAHCE was greater in males than in females in all age groups. Except for others, the CAHCE of neoplasms in females aged 65–75 years was the largest.

## Discussion

The purpose of this study was to test the “red herring” hypothesis established by Zweifel et al. [11] and to find the driving factors of end-of-life care costs by disease in Japan, where the population is aging more rapidly than in other OECD countries. In this study, we stratified 122,318 decedents aged 65 to 95 years who were enrolled in the NHI in the Shizuoka Prefecture, by age group and sex, and decomposed medical costs by disease group based on Bayesian methods. Frequency, IHCE, and AHCE had different profiles for each disease group for TTD, but the values tended to increase as the month of death approached. The profiles of frequency, IHCE, and AHCE for TTD differed among the categories stratified by age group and sex, but the differences among age groups were more pronounced than those by sex.

Wong et al. [24] analyzed the association between PTD and hospital HCE by primary disease and found that the effect of PTD was stronger in lethal diseases. They concluded that the effect of PTD was stronger in neoplasms, similar to other studies [21, 25]. In our analysis, the increase in IHCE of neoplasms with the approach of the month of death was large, but the same was also true for respiratory diseases. This may be because the total IHCE in this analysis was cost-allocated to each disease group in the Bayesian method, and costs were allocated to medical treatments that were not primary diseases, such as ventilator intubation near death. In Japan, the two major circulatory diseases, stroke and heart disease, account for approximately 30% of total deaths, and according to Wong et al.’s argument [24], the effect of PTD is expected to be stronger for circulatory AHCE. However, this was not the case in our analysis. This may be because many circulatory diseases are chronic, and their symptoms are controlled by continuous medication. Therefore, it is important to consider the medical treatment for each disease and its cost (reimbursement price) when examining the factors of HCE. These discussions were made possible by using the Bayesian method to allocate costs to disease groups other than the primary disease group, suggesting the importance of cost allocation.

Overall, in the results obtained from the Japanese data used in this analysis, AHCE was also larger as the month of death approached in each stratified category, which partially supported the “red herring” hypothesis proposed by Zweifel et al. [11, 30]. However, contrary to Zweifel et al.’s conclusion [30] that there was no effect of age on HCE among the deceased, AHCE differed among the age groups in the present study. In particular, AHCE and CAHCE in the age group of 65–75 years were larger than those in the age group of 85–95 years in all disease groups except for respiratory diseases, and the difference was especially pronounced in neoplasms. These suggest that there is an effect of both, PTD and age on HCE, which is consistent with several reports [15, 16, 18, 23, 32]. Hashimoto et al. [18] analyzed frequency and IHCE by inpatient and outpatient hospitalization in the year before death for individuals in the 65–75, 75–85, and 85+ years age groups in the Kyushu region of Japan, and reported that both frequency and IHCE were higher in younger age groups for almost all TTD for both inpatient and outpatient hospitalization. Meanwhile, in our study, frequency was higher in older patients with circulatory and respiratory diseases and higher in younger patients with neoplasms. Additionally, IHCE was larger in the younger age group with CKD and neoplasms and there was a large difference in the magnitude of the relationship between the age groups in each disease group. Although the trend of higher frequency in younger age groups was consistent in Hashimoto’s study for both inpatient and outpatient care, the age profile of frequency varied by disease in our study. This suggests that disease affects HCE more differently for each age group, and it is important to consider disease in the factor analysis of HCE. In addition, except for respiratory disease, AHCE and CAHCE were smaller in the older adults group, which may be due to a decrease in the intensity of inpatient care for them and a decrease in the hospitalization rate due to the use of nursing care [18, 22–24, 35].

Shugarman et al. [26, 36] analyzed the association between medical costs and age and gender among Medicare beneficiaries who died of lung cancer at age 68 years or older, and found that IHCE was greater in women than in men. In the present study, IHCE in the neoplasms group, including lung cancer, was higher in females across most TTD, consistent with the results of Shugarman’s study. However, IHCE in males was larger than that in females for respiratory diseases and others, and it is important to note that the relationship between the magnitude of the profiles of males and females differs by disease. In particular, in others, which includes the majority of the diseases, frequency was greater in females and IHCE was greater in males across many TTD. This trend is consistent with Hashimoto’s results [18]. Although women tend to be more likely to see a physician at the end of life, when men do see a physician, their illness tends to be more severe and their medical costs may be higher. As these indicate, it is important for estimating HCE before death to take into account the complex differences in frequency and IHCE by gender for each disease and TTD.

The impact of neoplasms on CAHCE, the medical cost in the 2 years prior to death, was greatest in the 65–75 age group. The CAHCE of females in the 65–75 age group was particularly large, with a reduction in medical costs of about 500,000 yen per person per year, even if the age at which the disease strikes is delayed by about 10 years. This is greater than the average annual medical cost for all Japanese people, which is approximately 350,000 yen [55]. To reduce the cost of end-of-life care, it is important to delay the onset of serious diseases as much as possible through preventive interventions such as lifestyle improvement and early detection and treatment in the category in which most medical resources are invested.

In this study, we proposed a method to estimate the average HCE of each disease group by cost allocation of HCE, which is aggregated on a monthly basis, to each disease group using a Bayesian method. We found that the relationship between HCE and age and sex differed in each disease group, and that the terminal care cost of neoplasms was relatively higher in the younger age group. However, because the sample size was not large enough for the Bayesian method to converge, it was difficult to allocate the costs to more detailed disease groups and inpatient and outpatient groups. This problem can be overcome by increasing the number of subjects in the analysis. In addition, we did not consider the fact that the distribution of medical costs is skewed, which may have caused bias. Furthermore, although we assumed that the IHCE of each disease group was independent of each other, the possibility of “super-additive,” in which the IHCE of comorbidities is larger than the sum of the independent IHCE of the underlying diseases, has been suggested [38], and this point may need to be considered. This analysis assumes that the relationship between age and sex and medical costs by disease group is stationary during the period of analysis, which is about 6 years. For example, in Japan, stroke incidence and mortality rates have been on a gradual downward trend due to changes in health status over time [56, 57], and the relationship between age and disease rates has not been completely stationary over time. Therefore, when estimating future medical costs, it may be necessary to incorporate dynamic incidence rates that take into account changes in health, economic, and social conditions over time, as seen in Kasajima’s study [58].

## Conclusions

In this study, using data from decedents enrolled in the Japanese NHI, we used a Bayesian approach to decompose the aggregated monthly medical costs into HCE for each disease group, and to examine the relationship between PTD and HCE by disease group, stratified by sex and age. As in recent studies, we found that HCE in most disease groups increased as death approached. However, the profiles differed greatly among disease, sex, and age groups, suggesting that they may be important driving factors for HCE. In addition, the large two-year cumulative medical cost of neoplasms in younger age groups suggests that preventive interventions such as lifestyle modification and early detection and treatment are important to reduce future medical costs in the end-of-life period. Not only for neoplasms, but also for other diseases that place a heavy burden on end-of-life care in the younger age group, the effect of delaying the onset of severe disease on reducing medical costs may be not negligible.

## Supplementary Information

**Additional file 1: Figure S1.** Frequency and IHCE (incurred health care expenditures) without stratification by sex and age group.

**Additional file 2: Figure S2.** AHCE **(**average health care expenditures) and CAHCE (cumulative average health care expenditures) without stratification by sex and age group.

**Additional file 3: Figure S3.** AHCE **(**average health care expenditures) and CAHCE (cumulative average health care expenditures) with stratification by age group and sex.

## Data Availability

The data that supporting the findings of this study were used under license for the current study from Shizuoka Prefecture in Japan. As such, they are not publicly available. However, data are available from the authors upon reasonable request and with permission from the Shizuoka Prefecture in Japan. (Shizuoka Prefecture, Japan: https://www.pref.shizuoka.jp/index.html).

## Abbreviations

AHCE: Average Health care expenditures
CAHCE: Cumulative average health care expenditures
CKD: Chronic Kidney Disease
HCE: Health care expenditures
IHCE: Incurred health care expenditures
MCMC: Markov Chain Monte Carlo
NHI: National Health Insurance
PTD: Proximity to death
TTD: Time to death
