# Supplementary figures and images for: Examining proximity to death and health care expenditure by disease: a Bayesian-based descriptive statistical analysis from the National Health Insurance database in Japan
Source: Health Econ Rev. 2022 Jan 10;12:6. doi: 10.1186/s13561-021-00353-9 (PMC8750752; doi:10.1186/s13561-021-00353-9)

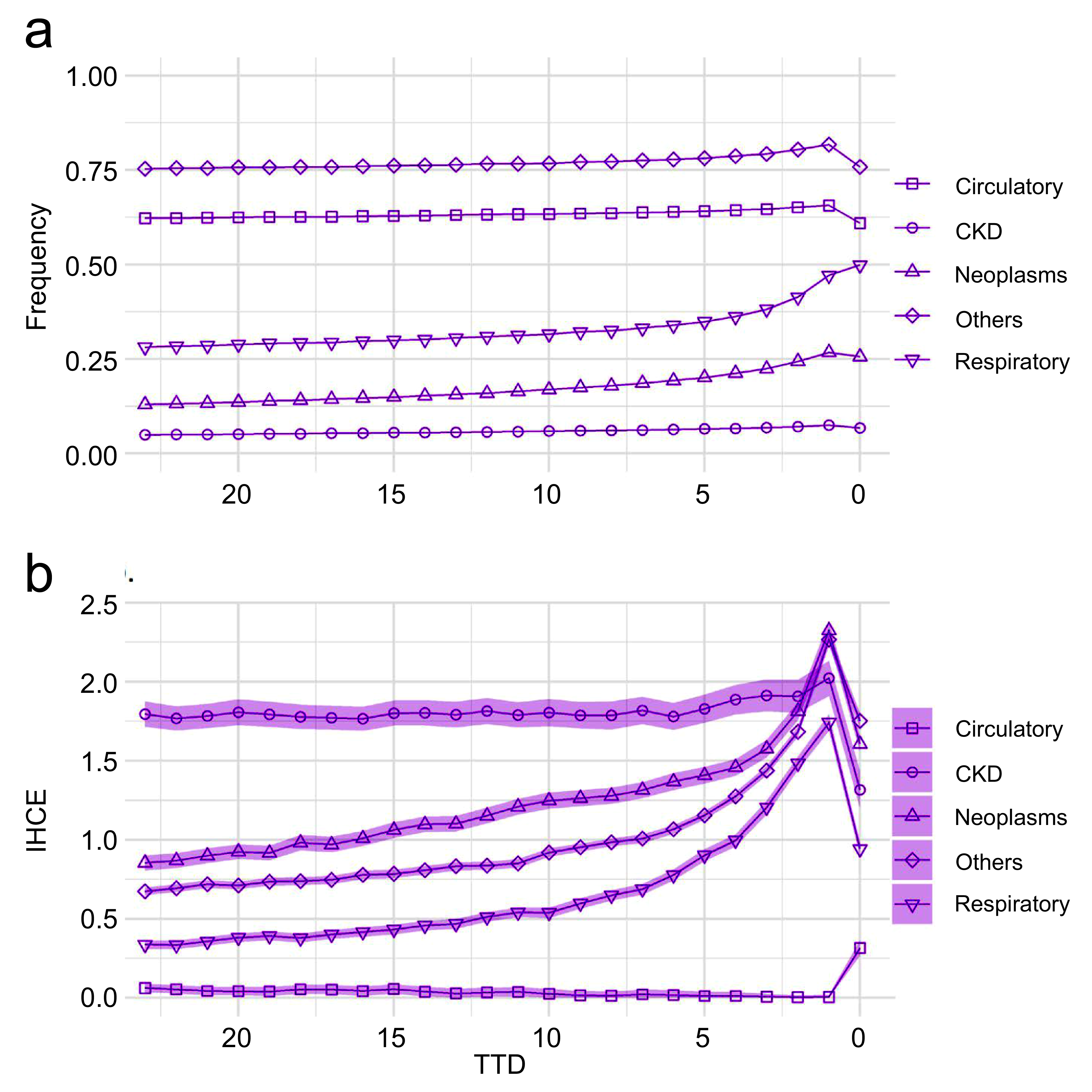

Supplement: Supplementary file 1 — Additional file 1: Figure S1. Frequency and IHCE (incurred health care expenditures) without stratification by sex and age group. [file 13561_2021_353_MOESM1_ESM.tif]

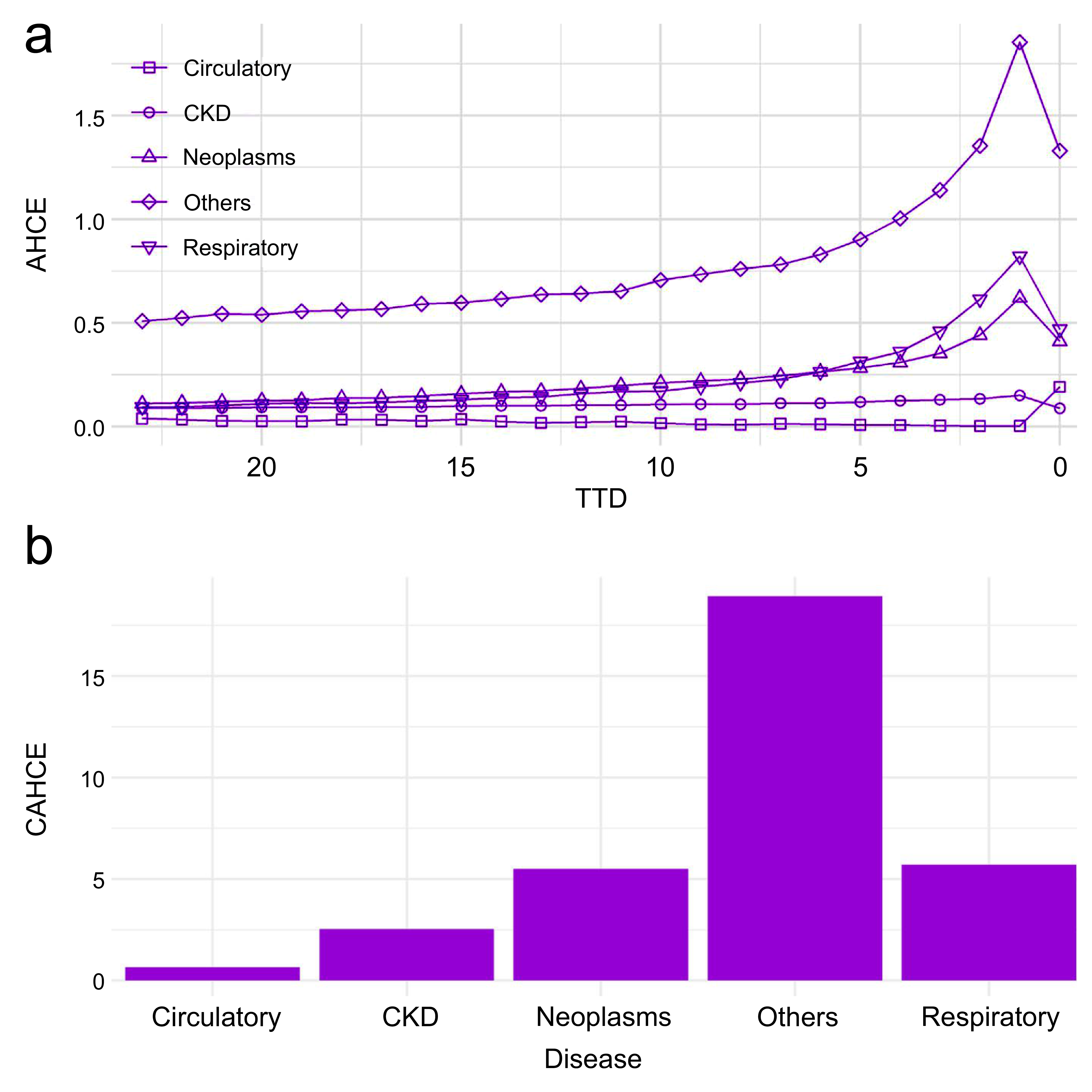

Supplement: Supplementary file 2 — Additional file 2: Figure S2. AHCE (average health care expenditures) and CAHCE (cumulative average health care expenditures) without stratification by sex and age group. [file 13561_2021_353_MOESM2_ESM.tif]

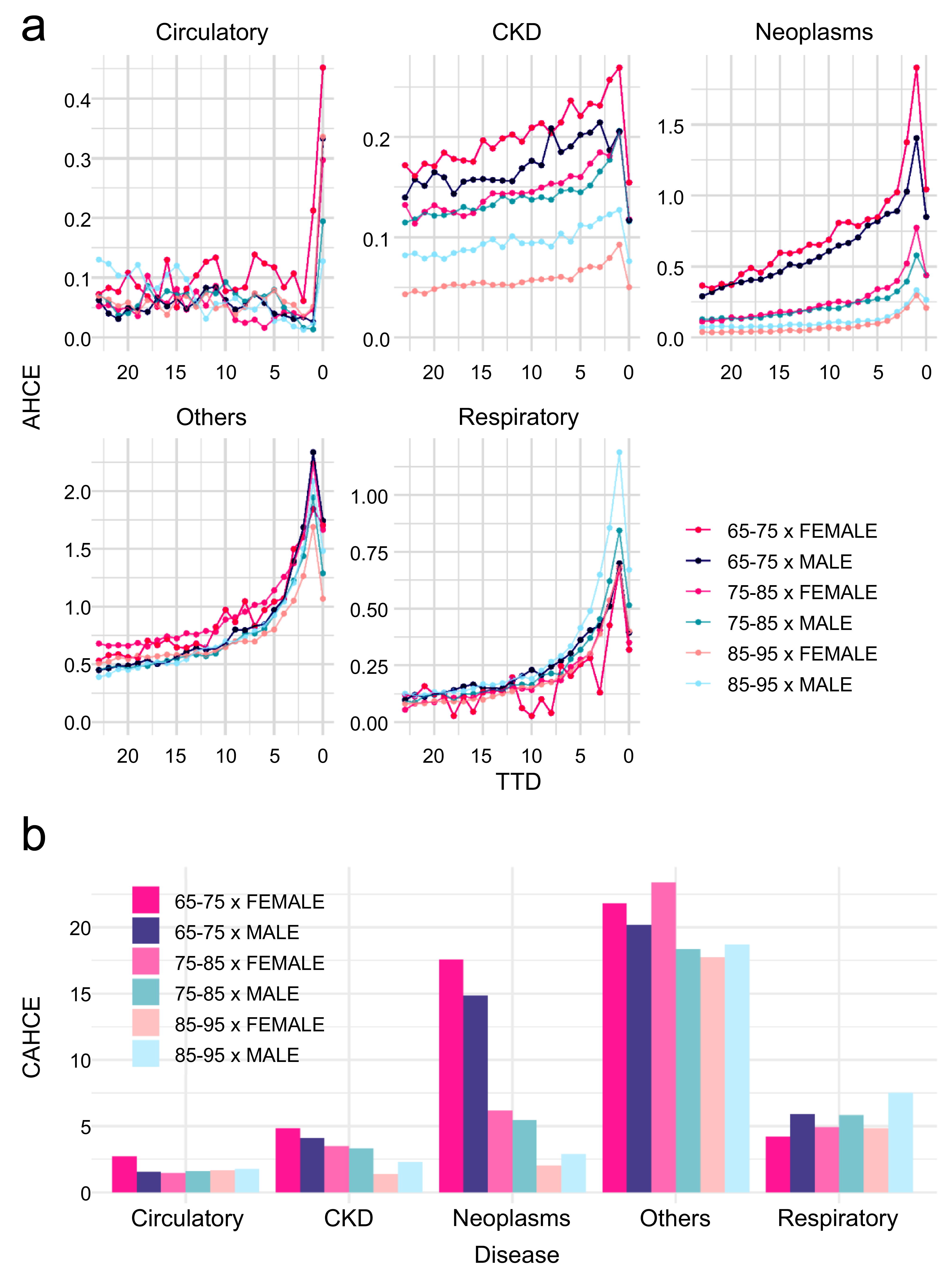

Supplement: Supplementary file 3 — Additional file 3: Figure S3. AHCE (average health care expenditures) and CAHCE (cumulative average health care expenditures) with stratification by age group and sex. [file 13561_2021_353_MOESM3_ESM.tif]
